# Quick Responses of Canadian Social Scientists to COVID-19: A Case Study of the 2020 Federal COVID-19-Specific Grant Recipients

**DOI:** 10.1007/s13753-022-00434-1

**Published:** 2022-08-25

**Authors:** Haorui Wu, Adele Mansour

**Affiliations:** 1 Canada Research Chair in Resilience, School of Social Work, Faculty of Health, Dalhousie University, Halifax, NS B3H 4R2 Canada; 2 School of Communication Science and Disorders, Faculty of Health, Dalhousie University, Halifax, NS B3H 4R2 Canada

**Keywords:** Canadian federal granting agencies, COVID-19, Quick response disaster research, Research projects, Research workforce, Social sciences

## Abstract

COVID-19 prompted an abundance of independent and collaborative quick response disaster research (QRDR) initiatives globally. The 2020 federal COVID-19-driven granting opportunities initiated the first official QRDR effort in Canadian history, engaging social scientists to rapidly address the pandemic-related societal influences. This study aims to portray the landscape of this nascent social science QRDR workforce through the first round of federal COVID-19-specific grant recipients. A case study approach was employed to analyze 337 social science projects with 1119 associated researchers, examining the demographic structure of these COVID-19-driven social science researchers and their research projects’ characteristics. Accordingly, the findings are presented through the following two streams: (1) From a researcher perspective, this case study describes researcher typology, geographic location, primary discipline, and educational background, highlighting the diverse characteristics of social sciences researchers, and uneven research development across Canada. (2) From a research project perspective, this case study identifies and synthesizes research project subjects, themes, collaborations, and Canadian distinctions, emphasizing the need for galvanizing cooperation and focusing on uniquely Canadian contexts. The case study illustrates challenges associated with data curation that pose barriers to developing a nuanced understanding of the Canadian social science community COVID-19 research landscape. Consequently, the case study develops three recommendations to improve QRDR development in Canada: promoting information transparency, dissemination, and updates; improving hazards and disaster research workforce evaluation; and enhancing multi-stakeholder cooperation.

## Introduction

COVID-19 prompted remarkable global independent and collaborative quick response disaster research (QRDR) initiatives (Nature 2020). The QRDR provides time-sensitive insights into disaster impacts, collecting “perishable” or “ephemeral” aftermath data “before memories fade and physical evidence is erased” (NHC n.d.a, para. 1). A worldwide dramatic rise in disaster-caused human, economic, and social losses propelled many countries to establish national QRDR programs (Australian Government n.d.; NSF n.d.; Evanson and Scheuber 2019). Although Canada has entered an era of frequently occurring, billion-dollar disasters (Public Safety Canada 2015), its national QRDR program has been slow to be established. The three Canadian federal granting agencies (referred to as Tri-agencies), namely the Canadian Institutes of Health Research (CIHR), the Natural Sciences and Engineering Research Council (NSERC), and the Social Sciences and Humanities Research Council (SSHRC), primarily support research, training, and innovation in postsecondary organizations across Canada (Government of Canada 2021a). In response to the global COVID-19 crisis, CIHR (2020a) and SSHRC (2020) launched COVID-19-specific QRDR grants in February and April 2020, respectively. These grant competitions were considered the first federal QRDR initiatives launched in Canada. In addition to medical and health impacts, the full COVID-19-specific societal influence spectrum drives social scientists to investigate various vulnerabilities at the individual, family, community, and societal levels, with the aim to build resilient and sustainable societies in Canada and beyond (Wu and Karabanow 2020).

Historically, social scientist-led QRDR offers community-based knowledge, skills, and strategies to promote current hazards and disaster research and inform related disaster and emergency management practice and policy (Tierney 2015). Understanding the demographic structure of social scientist-led QRDR enables the swift coordination of local professionals to respond to their community-based extreme events (National Research Council 2006). Consequently, since 2018, the NSF-funded Social Science Extreme Events Research (SSEER) project, led by Dr. Lori Peek, director of the Natural Hazards Center (NHC) at the University of Colorado Boulder, has been conducting the first-ever census of social science hazards and disaster research workforce in the United States and internationally (Peek, Champeau, et al. 2020). Since the census has been processed through U.S.-based networks, the Canadian data collected in the SSEER project cannot precisely reflect the Canadian social science research workforce. Furthermore, as mentioned above, the Tri-agency COVID-19 grants have launched the QRDR initiatives in Canada, and the societal impacts of COVID-19 stimulate the Canadian social scientists’ engagement. Through a systematic review of U.S. QRDR programs and reports, Oulahen et al. (2020) suggest that developing a Canadian-driven quick response social science research program would not only accurately identify diverse Canadian community-specific disaster impacts, but more importantly, fulfill the Canadian disaster science sub-disciplinary QRDR program deficit. How have the social scientist-led COVID-19 QRDR efforts improved the Canadian QRDR deficit?

In response to the research deficits identified by Peek, Champeau, et al. (2020) and Oulahen et al. (2020), this research has employed a case study approach, with the aim of understanding the nascent social science QRDR research workforce in the 2020 federally funded, COVID-19-specific QRDR initiatives in Canada. This case study investigates the demographic structure of the social scientists and their projects’ characteristics. This understanding will provide evidence-based strategies to coordinate and strengthen the Canadian social science QRDR workforce, improve Canadian social scientist-led QRDR development, and promote multidisciplinary, multi-stakeholder QRDR engagement in Canada and internationally.

## Quick Response Disaster Research in Social Science Hazards and Disaster Research Community

Hazards and disaster research has a well-developed history in engineering and natural science-related disciplines (Tierney 2019). Multidisciplinary and multi-stakeholder hazards and disaster research, practice, and management inherently embrace an inter-/trans-disciplinary approach to create a foundation for building resilient and sustainable communities (Peek, Tobin, et al. 2020). Social scientists essentially contribute to this inter-/trans-disciplinary approach through theoretical frameworks, methodologies, promising practice, and policy/decision making (Peek and Guikema 2021). In this case study, the social sciences’ definition is rooted in the interplay between humans and society, reflecting a series of social science disciplines described by Laidlaw et al. (2020). The following section will discuss Canadian QRDR development, the affiliated research workforces, and their various foci, in order to scaffold this case study’s rationale and importance.

### Quick Response Disaster Research Development in Canada

Social science QRDR unveils extreme event aftermath interactions among human beings, co-inhabitants (animals), and communities (NHC n.d.a; Morris et al. 2021). Based on time-sensitive data, QRDR outcomes provide valuable references, not only promoting disaster science development but also formulating community-based strategies for disaster risk reduction in various societal dimensions (Wu, Perez-Lugo, et al. 2021). The current upward trajectory of global extreme events, to some extent, has encouraged swift QRDR growth and advancement. Specifically, with NSF financial support, the NHC has successfully operated a QRDR program for over 36 years (since 1986), with more than 300 QRDR reports covering various disasters, spanning from the 1986 California floods, to Hurricane Katrina (2005), to the current COVID-19 pandemic (NHC n.d.b). These projects have quickly deployed researchers and other professionals into disaster-affected communities to collect perishable data and support emergency response initiatives. More significantly, these research projects provide tangible training and mentoring opportunities for next-generation hazards and disaster researchers and practitioners (CONVERGE n.d.) while creating networks among researchers, local authorities, affected communities, and their residents. These extensive benefits promote knowledge dissemination, translation, and mobilization, promoting ongoing post-disaster reconstruction and recovery of affected communities, regions, and beyond.

Although the Tri-agencies did not have QRDR grants before COVID-19, there were some research organizations across Canada that financially supported QRDR-related initiatives. Specifically, the Institute for Catastrophic Loss Reduction (ICLR), affiliated with Western University (Ontario, Canada) and the most prestigious Canadian multidisciplinary disaster prevention research and communication hub, launched Canada’s first QRDR program in 2016 (ICLR n.d.a). This QRDR program focused on the Albertan Fort McMurray wildfires, the costliest disaster in Canadian history so far (Magill 2016). The ICLR also collaborated with international (that is, NHC) and Canadian (that is, the Marine Environmental Observation, Prediction, and Response Network) research institutions to promote their QRDR agenda. The ICLR-led QRDR initiatives comprehensively explore municipal-level catastrophic disaster impacts pertaining to Canada-specific extreme events, such as wildfires and floods (ICLR n.d.b). These preliminary efforts promoted Canadian QRDR development and federal and provincial support that would significantly advance Canadian hazards and disaster research communities.

The global COVID-19 pandemic has significantly damaged human health and well-being, societies, and economic development worldwide (United Nations Environment Programme and International Livestock Research Institute 2020). Since the onset of COVID-19 in 2019, the CIHR, whose mandates are congruent with this public health emergency, has encouraged Canadian researchers to collaboratively decipher COVID-19-specific challenges and pursue related solutions (CIHR 2020a). Although the CIHR typically finances medicinal and health studies, the diverse COVID-19-driven societal impacts have propelled CIHR to create COVID-19-specific granting opportunities across diverse health and social dimensions. Collaborating with other federal granting and research agencies, on 10 February 2020, CIHR (2020b) launched the first COVID-19-specific QRDR grants in two areas: “medical countermeasures research” and “social and policy countermeasures research in health” (CIHR 2020a, para. 3). This competition launched the first official QRDR grants in Canada: 227 research proposals involving 1,195 researchers were submitted within a 9-day application window, and 150 peer reviewers across Canada completed reviews within 5 days (CIHR 2021a). This expedited granting process ensures sufficient time to support research efforts that address urgent needs with the collection of time-sensitive data. As COVID-19 rapidly evolved, CIHR released five additional rounds of COVID-19-specific QRDR grants to generally attend to emerging COVID-19-driven health and social issues (CIHR 2020c), and more particularly, address medicinal challenges, such as clinical epidemiology (CIHR 2020d), mental health, and substance use (CIHR 2020e).

Comparatively, in April 2020, SSHRC (2020) launched two-round COVID-19-specific QRDR grants through Partnership Engage Grants (PEG), with application deadlines of 15 June and 15 September 2020, respectively (SSHRC 2021a). Generally, PEG encourages social sciences and humanities academic researchers to collaborate with public, private, or not-for-profit sectors to develop agency-driven evidence that would improve agency operation (SSHRC 2021a). These six-round CIHR competitions and two-round SSHRC grants are foundational in establishing Canadian-driven QRDR within the Canadian hazards and disaster community and stimulating other Canadian federal and provincial granting agencies’ initiatives in supporting QRDR.

### Social Science Quick Response Disaster Research Efforts

Global climate change, disaster, and other worldwide crises are prompting nations, worldwide, to develop community-driven strategies for climate change adaptation and disaster risk reduction. However, the Canadian national climate adaptation strategy is still developing (Boisvert 2021), even though Canadian climate change is accelerating at double the global average, and, imperatively, Northern Canada is warming three times faster than the global average (Government of Canada 2021b). Furthermore, countries, such as the United States (Ramirez and Thompson 2019) and the United Kingdom (UKCRIC 2019), have been focusing on hazards and disaster research workforce and resource evaluations, so they can swiftly coordinate their physical, social, and human resources in response to extreme events at the local, regional, and national levels. Notably, as discussed above, SSEER has been using surveys to identify global hazards and disaster social science researchers (Peek, Champeau, et al. 2020). As 2021 concluded, SSEER’s database comprised 1420 researchers worldwide (NHC n.d.c). The U.S.-based nature of SSEER indicates that American data would be more accurate than other countries. For example, the current SSEER map (2022) shows that there are 58 social scientists located in six Canadian provinces (NHC n.d.d). This number might not comprehensively reflect the entire Canadian social science hazards and disaster research community; however, no hazards and disaster research workforce datasets are available in Canada, let alone those serving in social sciences and humanitarian-related disciplines.

The COVID-19 pandemic has compelled global multidisciplinary research communities to independently and collectively fight a common enemy (WHO n.d.). Worldwide COVID-19 research projects formulate a valuable dataset to support COVID-19-specific QRDR research workforce investigation at home and abroad. Particularly, Columbia University (2021) leads the U.S. COVID Information Commons initiative, analyzing 1722 NSF-funded COVID-19 rapid response research projects. This study promotes collaboration and knowledge dissemination among students, researchers, practitioners, decision makers, and other stakeholders in public, private, and not-for-profit organizations in the United States and beyond (Columbia University 2021). In Canada, CanCOVID (2021), a federally-funded networking platform, has opened a data-sharing platform among Canada-wide COVID-19 studies. This platform includes most medical and health research projects; however, social sciences and humanities projects have not been effectively presented. Moreover, COVID-19 Resources Canada (n.d.) utilizes web-based data collection concerning researchers and their projects. Data collection depends on the researchers’ voluntary input and therefore cannot portray a comprehensive Canadian researcher landscape. The common challenge for these agencies’ data is that the researchers’ academic information (for example, their disciplinary expertise and geographic locations) is unclear.

Social scientists’ community-driven expertise directly addresses the pandemic’s catastrophic impacts on local and international communities (Reinhardt and Ross 2019). Social science QRDR thoroughly explores specific population group disaster-driven vulnerabilities, typically pertaining to vulnerable and marginalized groups, such as people experiencing homelessness (Karabanow et al. 2022) and Indigenous populations (Stukes and Wu 2020). Since COVID-19-related risks are inherent to frontline workers, more atypical vulnerable groups, such as human and animal healthcare professionals (Mitchinson et al. 2021; Baysinger and Kogan 2022), have also been examined in this context. Social science QRDR also examines disaster-triggered societal impacts, such as social isolation and mental health (Johnson et al. 2021), work-family conflicts (DesRoches et al. 2021), and human-animal bonds (Wu, Bains, et al. 2021). These social science efforts fundamentally empower human dignity, advocate human rights, and promote environmental and social justice, contributing to resilient and sustainable communities (Drolet et al. 2015). Collecting community-driven data could help community-based service agencies and decision makers obtain a better understanding of the unique community needs, and subsequently customize community-based services and policies to address unique population-specific needs (Doll et al. 2022). In the COVID-19 context, these data are vital for social science QRDR. As global evidence demonstrates, swiftly coordinating community-based researchers and research teams to address unique community needs is fundamental to various pre-, peri-, and post-disaster efforts (Beaven et al. 2019). As aforementioned, to the best of the authors’ knowledge, no existing research initiatives focus on these dimensions through Canadian COVID-19-specific QRDR.

Succinctly, the research deficits presented above prompt a need to scan Canadian social scientist QRDR agendas in response to COVID-19. The two Canadian federal granting agencies’ (CIHR and SSHRC) COVID-19-specific QRDR grants award lists enable an initial analysis of the demographic structure of the social science research workforce and their research projects’ characteristics. This case study concentrates on CIHR and SSHRC’s dataset and is guided by the following research question: How did the Canadian social science QRDR community respond to COVID-19?

## Case Study

As the primary research funding resource, Tri-agencies designated grants have become the first choice for most Canadian postsecondary institution-affiliated researchers (McGill University n.d.; University of Windsor n.d.). Accordingly, a Tri-agencies funded research project’s case study enables an initial understanding of the Canadian QRDR workforce. Furthermore, among the three federal agencies, social sciences research initiatives were primarily financed through CIHR and SSHRC programs rather than NSERC programs (NSERC n.d.). The CIHR and SSHRC grant competition results constitute the primary data resources of this case study.

### Initial Data Curation and Screening

Among the COVID-19-specific QRDR call for proposals released by the Tri-agencies in 2020, there are eight rounds of federal grants in Canada, namely six CIHR grants and two SSHRC grants. Among the six CIHR grants, the two-round general grants funded 253 projects; specifically, there were 100 projects funded in the first call and 153 in the second one. The remaining four-round specific grants awarded 28 projects. Hence, six rounds of CIHR QRDR initiatives funded a total of 281 projects. Project titles, abstracts, keywords, and research teams are available on the CIHR funding decisions notifications (CIHR n.d.). The first-round SSHRC competition funded 172 projects (in September 2020), and the second-round competition funded 123 projects (in December 2020), with a total of 295 projects. Compared to CIHR funding competition notifications, SSHRC awards recipients comprise the names of researchers, their affiliated organizations, partner organizations, project titles, and the amount of funding, but do not include project abstracts and keywords as the CIHR funding decision notifications released (SSHRC 2021b, 2021c).

**Fig. 1:**
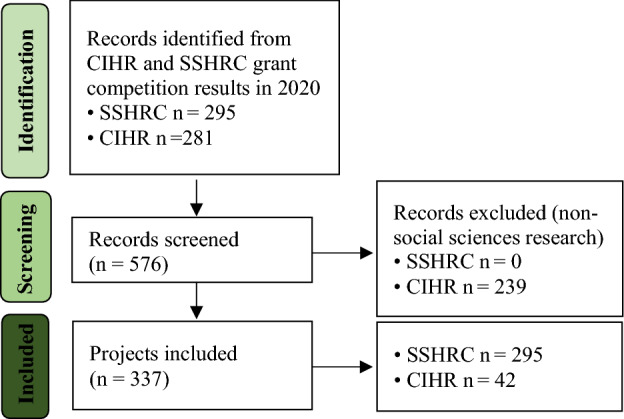
Flowchart identifying the COVID-19-specific social scientist-led quick response disaster research projects in Canada. CIHR: Canadian Institutes of Health Research; SSHRC: Social Sciences and Humanities Research Council. *Source* Adapted from Page et al. (2021).

As described above, the initial database consists of all CIHR and SSHRC-funded COVID-19-specific QRDR projects. The two authors with interdisciplinary social and health research backgrounds independently reviewed the 576 project titles and abstracts (CIHR-funded projects only) and discussed to identify social science research projects. As CIHR-funded ventures are primarily medicinal and health research focused, only 42 of the 281 projects were classified as social science research (that is, medically-focused projects, such as vaccine development, and physical health injury diagnostic tests were removed). While the two-round SSHRC calls were explicitly designated as COVID-19-related social and humanitarian research streams, all these SSHRC-funded projects were included in the final dataset. As shown in Fig. 1, there were 337 social-scientist-led COVID-19-specific projects, with 295 SSHRC-supported and 42 CIHR-supported.

### Further Data Curation and Analysis

As most grant recipients are affiliated with Canadian postsecondary institutions, the authors used the grant awardees’ names and associated organizations to search their professional webpages and collected related information, such as primary discipline, career stage, and highest degree obtained. Furthermore, Canadian postsecondary organizations and public media also published news to introduce these funded projects, providing the necessary details to understand the projects’ content, particularly relevant for SSHRC-funded projects where abstracts are not publicly available. The authors also searched grant recipients’ current publications that were dated after their projects’ release, such as peer-reviewed journal articles, op-eds, popular media articles, reports, briefs, and other forms of grey literature. The grey and academic literature assisted the research team in understanding the basic information of funded research projects and ultimately supporting the data analysis. However, this extra information (such as news, website content, and academic and grey literature) was unavailable for every awardee, triggering potential challenges for this case study. These challenges and related limitations will be discussed later in this article.

The final dataset includes project titles, researcher names, affiliated organizations, home departments (for university or college-based researchers only), primary disciplines, project themes, research team composition, and so on. The final dataset can be accessed from Mansour and Wu (2021). Due to privacy and ethical concerns, individual data (namely, names, e-mail addresses, and office telephone numbers) were removed from this published dataset. Data analysis engaged a quantitative approach (that is, frequency and percentage) for the demographic structure of the research workforce and a qualitative approach (that is, coding and theming) for research projects’ characteristics. The quantitative and qualitative analyses were supported by SPSS Statistic and NVivo 12, respectively. The two researchers independently analyzed the data, conducted weekly discussions, and synthesized the final findings that are presented in the next section.

## Findings

This case study illustrates the 2020 Canadian COVID-19-specific social science QRDR landscape. As Table 1 demonstrates, through 337 QRDR projects, a total of 1119 researchers (including principal investigators, co-investigators, collaborators, and knowledge users) have individually and collaboratively (up to 20 researchers in one team) examined diverse COVID-19-specific societal impacts, at the individual, family, community, and societal levels. Among these research teams, 1005 researchers are affiliated with SSHRC-funded projects, and 114 are affiliated with CIHR-funded projects.

**Table 1:** General statistics of the COVID-19-specific social scientist-led quick response disaster research projects in Canada

	SSHRC	CIHR	Total
Number of projects	295	42	337
Number of researchers	1005	114	1119
Number of international collaboration projects	5 (1.7%)	0 (0%)	5 (1.5%)
Number of interprovincial collaboration projects	60 (20%)	14 (33%)	74 (21.9%)

*CIHR* Canadian Institutes of Health Research, *SSHRC* Social Sciences and Humanities Research Council

COVID-19 catastrophically impacts communities domestically and internationally. These broader effects have also been reflected in Canada’s COVID-19-specific QRDR agenda. Among the 337 projects, there are five international partnership projects, all funded by SSHRC. At the national level, 74 projects (60 SSHRC-funded projects and 14 CIHR-funded projects) feature interprovincial partnerships. Interprovincial collaboration eligibilities are determined according to the following two criteria: Criterion 1: at least two research team members’ primary affiliations are located in two jurisdictions in Canada; Criterion 2: if the entire research team is located in the same jurisdiction, their studies must be conducted outside of this jurisdiction. The interprovincial collaborative projects represent 20% of SSHRC-funded projects (60 projects in total) and 33% of CIHR-funded projects (14 projects in total). The following sections provide a detailed twofold analysis: the demographic structure of the social science research workforce and their research projects’ characteristics.

### Researchers

This section examines the demographic structure of the social scientists from the following four aspects: typology, geographic location, primary discipline, and educational background. Understanding these four variables will contribute to strengthening and coordinating the Canadian social science QRDR workforce to swiftly respond to extreme events in the future.

#### Typology

Generally, based on the frequency, depth, and scope of researchers’ engagement in the hazards and disaster field, four typologies were developed, namely, core researcher, periodic researcher, situation researcher, and emerging researcher (Peek, Tobin, et al. 2020); For example, core researchers claim deep engagement in this field, and hazards and disaster research represents the central part of their entire research agenda while emerging researchers are new to this field and are presently building their research experience (Peek, Tobin, et al. 2020). This typology-specific information requires self-identification, which could not be detected through an Internet-based search approach. Nevertheless, the Tri-agencies use emerging and established scholars to describe Canadian researcher typologies and established specific grant streams to assist the early career (emerging) researchers in building their research achievements (Government of Canada 2020). Specifically, federal funding agencies mainly support researchers affiliated with Canadian postsecondary organizations; emerging scholars who are situated within 6 years of the completion of their highest degrees (usually a doctoral degree or equivalent) or within the 6-year threshold of their tenured or tenure-track postsecondary appointment, including postdoctoral fellow and other research associates, instructors, assistant professors, and some associate professors (SSHRC 2021d). The established researchers have recognized research records and achievements and are mostly associate and full professors at research-intensive universities across Canada (SSHRC 2021d).

Furthermore, the Tri-agencies require principal investigators to be affiliated with eligible Canadian institutions, where postsecondary institutions (universities and colleges) and affiliated research organizations (such as hospitals and laboratories) occupy most of these eligible institutions (SSHRC 2021e). The principal investigators named in these 337 projects are all affiliated with postsecondary institutions and/or affiliated research organizations. Other postsecondary institution-affiliated professionals (that is, librarians and research support staff), who make significant contributions to the research project, serve as co-investigators or collaborators. The SSHRC COVID-19-specific grants were offered through the Partnership Engage Grants, which aim to encourage academic researchers to collaborate with public, private, or not-for-profit sectors to develop agency-driven evidence to improve agency operation (SSHRC 2021a). Hence, researchers from these non-academic agencies were involved in these projects, contributing to research team composition.

**Fig. 2:**
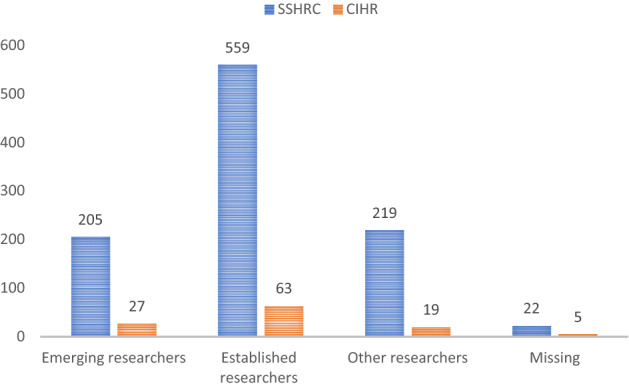
Researcher typology of the COVID-19-specific social scientist-led quick response disaster research projects in Canada. CIHR: Canadian Institutes of Health Research; SSHRC: Social Sciences and Humanities Research Council.

Considering researcher characteristics, Fig. 2 illustrates the researcher typology in this case study. Although these SSHRC and CIHR grants did not provide priority to emerging scholars, it can be clearly discovered that the percentage of emerging scholars among the total researchers is solid. Remarkably, the ratios of emerging and established scholars are 1: 2.7 among SSHRC-funded projects and 1: 2.3 among CIHR-funded projects.

#### Geographic Locations

The two authors used researchers’ primary affiliated institutions to identify their geographic locations. Among the 1005 researchers who conducted SSHRC-funded projects, 935 are Canadian researchers (93%) and 70 come from overseas (7%). The CIHR-funded projects show that Canadian researchers are the majority, at 96.5% of total researchers (110 Canadian researchers and four international researchers). The 74 international researchers from both the CIHR-funded and the SSHRC-funded projects represent 14 nations, including the United States, the United Kingdom, Israel, China, Sweden, South Africa, Tanzania, and Peru.

**Fig. 3:**
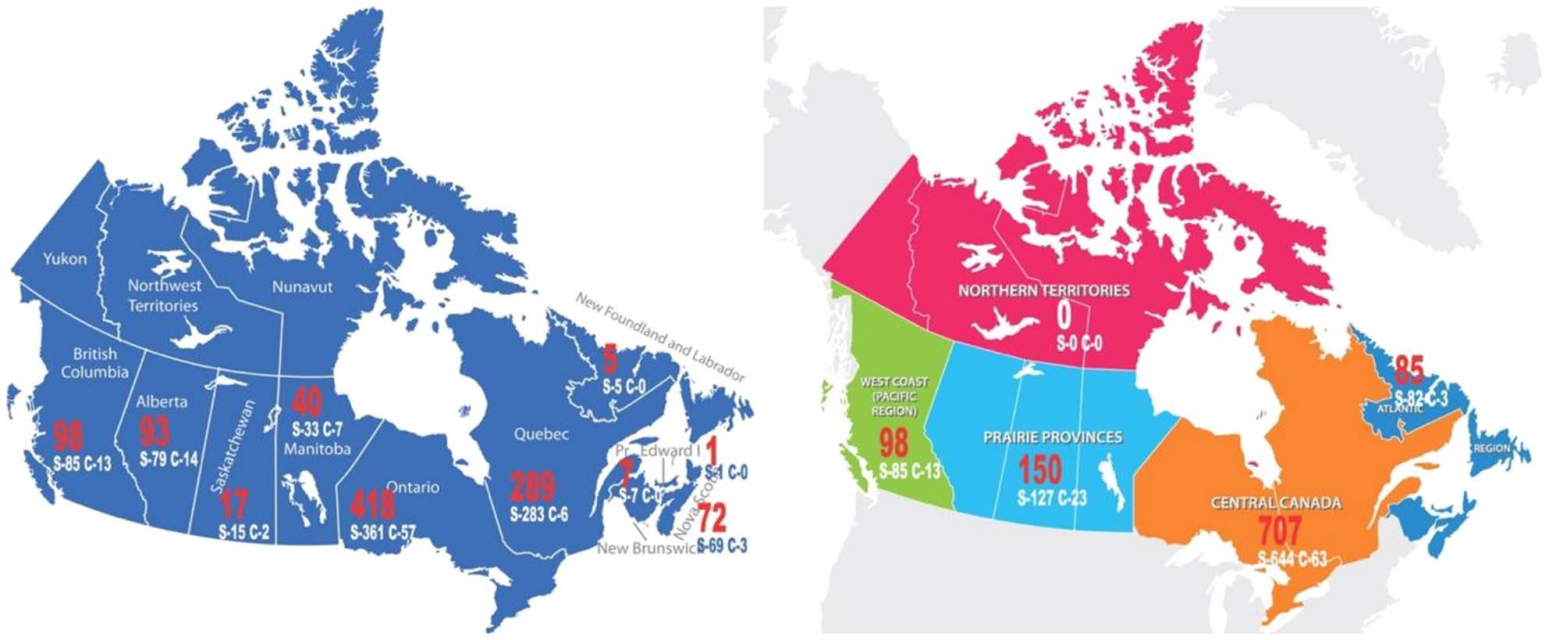
Geographic locations of researchers. Left: provincial locations. Among the 10 Canadian provinces, the red numbers show the total number of funded researchers in each province, broken down by the two funding agencies, SSHRC (Social Sciences and Humanities Research Council) and CIHR (Canadian Institutes of Health Research), respectively. Right: regional locations. This regional map shows the five regions in Canada. The red numbers show the total number of funded researchers in each region, broken down by the two funding agencies, SSHRC and CIHR, respectively.

The current SSEER map shows 58 social scientists located in six Canadian provinces (NHC n.d.d). The researchers engaged in this case study provide complementary SSEER information, and promote up-to-date social scientist research workforce understanding associated with COVID-19. The province-based social scientist number is visualized on the national map (Fig. 3). The analysis results are presented at the federal, regional, and provincial levels. Please note that both maps show 1,040 Canadian researchers, respectively. There are 74 international researchers, who are excluded from the Canadian maps, and five researchers, whose geographic locations are unknown.

National-level observation: The map shows that COVID-19-driven researchers are located across the 10 Canadian provinces. Unfortunately, no researchers are found in the three northern Canadian territories (Yukon, Northwest Territories, and Nunavut), reflecting Canada’s uneven educational and research resource allocation, as these territories have the fewest postsecondary institutions in Canada (Yuen-Yung and Wu 2021).

Regional-level observation: Across Canada, 10 provinces and three territories are grouped into five distinct regions (from east to west and from south to north), the Atlantic Provinces (New Brunswick, Newfoundland and Labrador, Nova Scotia, and Prince Edward Island), Central Canada (Ontario and Quebec), the Prairie Provinces (Alberta, Saskatchewan, and Manitoba), the Pacific (British Columbia), and the Northern Territories (Yukon, Northwest Territories, and Nunavut). This unequal researcher distribution shows that no researchers are located in the Northern Territories, while the Central Region bears the most researchers (707 researchers), which is about 8.3 times greater than the Atlantic Region (85 researchers). This unequal distribution reflects the previously noted national-level educational resource disparities.

Provincial-level observation: The top three provinces where the most researchers are located are Ontario (418 researchers), Quebec (289 researchers), and British Columbia (98 researchers). Undoubtedly, these three provinces have the most significant number of Canadian universities and colleges. According to Times Higher Education (THE n.d.) 2022 world university rankings, the top three Canadian universities, the University of Toronto (Ontario), the University of British Columbia, and McGill University (Quebec), are among the internationally top 50 educational institutions (THE n.d.). These domestic and international rankings are aligned with the researchers’ census data in this study. The three provinces with the smallest number of researchers are New Brunswick (7 researchers), Newfoundland and Labrador (5 researchers), and Prince Edward Island (1 researcher), all situated in the Atlantic Region.

#### Primary Disciplines

**Fig. 4:**
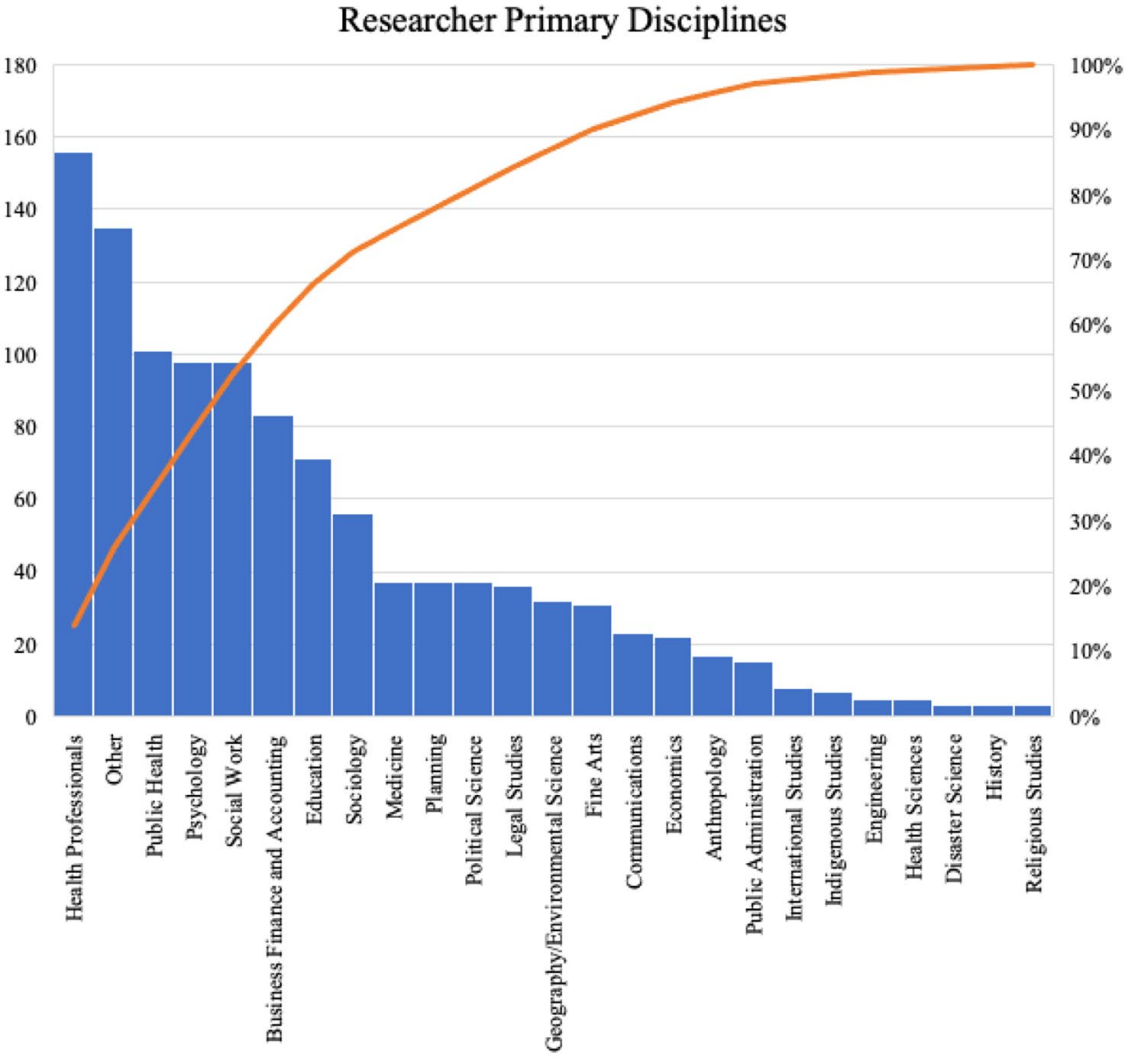
Researcher primary disciplines of the COVID-19-specific social scientist-led quick response disaster research projects in Canada. Left axis: the number of researchers; Right axis: cumulative percentage.

Generally, researchers’ professional webpages on affiliated institutional websites indicate their primary disciplines, which were collected in this case study. Unavailable researcher primary discipline information is shown as “other” in Fig. 4. Health-related disciplines, such as health professionals (that is, nursing and pharmacy), public health, psychology, and social work, represent the majority of researcher disciplines, which are aligned with the nature of COVID-19-specific research. Other social science disciplines, including business, finance and accounting, education, sociology, and religious studies, further contribute to COVID-19-related multidisciplinary engagement.

#### Educational Backgrounds

Searching researchers’ professional web pages revealed related professional information, such as educational background (highest degree). As indicated above, all the 295 SSHRC-funded projects required agencies as collaborators. The researchers from these agencies might not list their educational backgrounds and/or their demographic factors (such as gender and ethnicity) on their agencies’ websites. Hence, this case study only reports the researchers’ highest degrees. As shown in Table 2, 81.6% of the researchers hold doctoral or equivalent degrees. This number is significantly higher than the Peek, Champeau, et al. (2020) census result (62.59%).

**Table 2:** Researchers’ educational attainment of the COVID-19-specific social scientist-led quick response disaster research projects in Canada

Highest degree	SSHRC		CIHR		Total	
	Frequency	%	Frequency	%	Frequency	%
PhD/JD/MD	814	81.0	99	86.8	913	81.6
Masters	61	6.1	5	4.4	66	5.9
Bachelors	19	1.9	1	0.9	20	1.8
Diploma	1	0.1	1	0.9	2	0.2
Missing	110	10.9	8	7.0	118	10.5

*CIHR* Canadian Institutes of Health Research, *SSHRC* Social Sciences and Humanities Research Council

### Research Projects

In addition to researcher names and affiliated organizations, the original awarder list data included research project titles, which present basic project content such as research subjects and themes. The grey literature, such as research news published by the researchers’ affiliated organizations, also briefly introduced the awarded projects. This additional information enabled the research team to analyze and synthesize some critical characteristics of these funded research projects.

#### Research Subjects

**Fig. 5:**
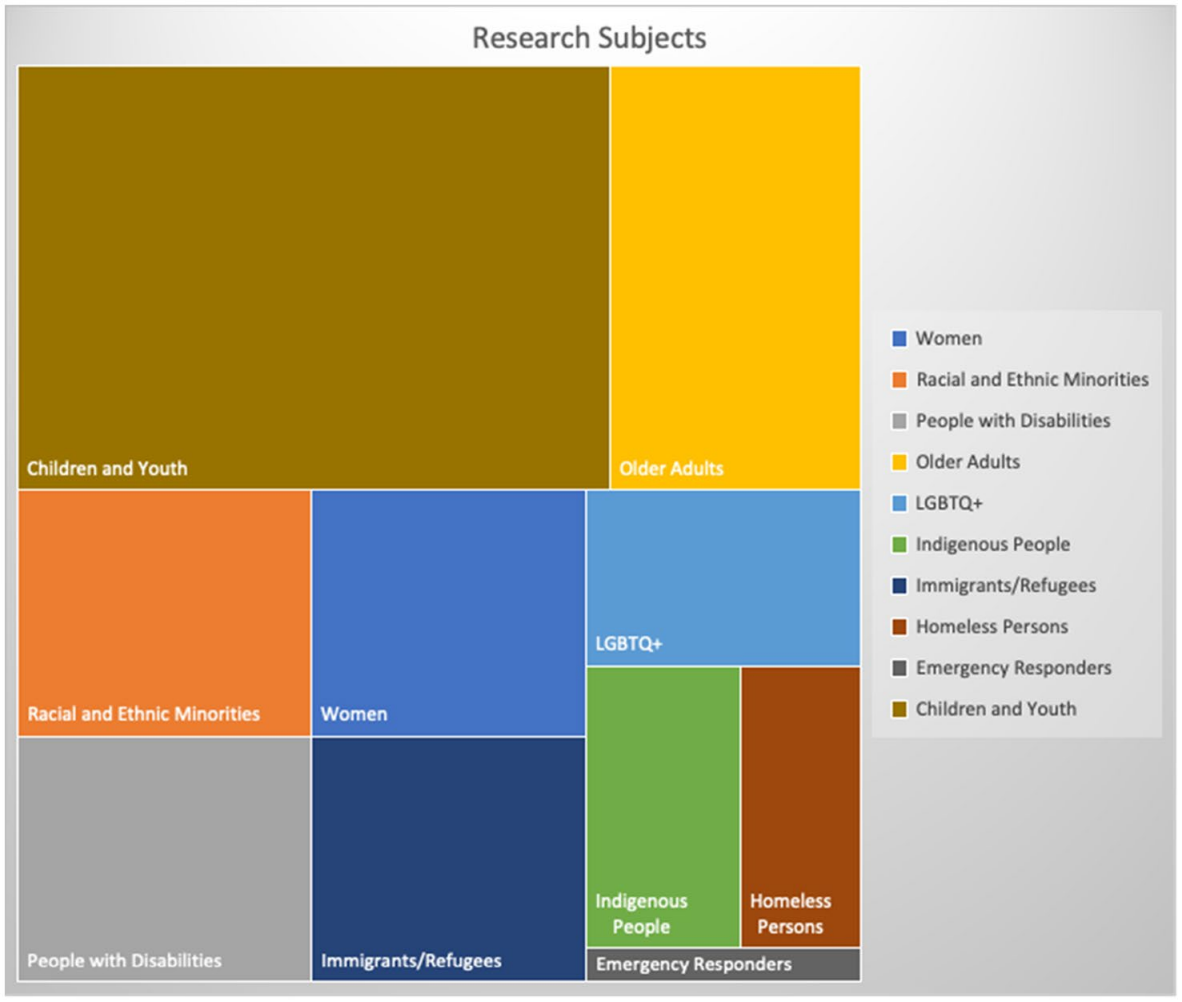
Research subjects of the COVID-19-specific social scientist-led quick response disaster research projects in Canada

While COVID-19, like other extreme events, affects the entire population, it disproportionately impacts vulnerable and marginalized groups (Karabanow et al. 2022). Building resilience capacity and advocating for vulnerable and marginalized groups’ basic living and social rights have become an integral theme in hazards and disaster research and practice, particularly for social scientists (Peek, Champeau, et al. 2020; Wu et al. 2022). Of the 337 COVID-19-specific QRDR projects, 177 project subjects were not clearly identifiable from the available information derived from project titles and related news releases. Figure 5 depicts the remaining 160 projects’ identifiable research subjects. The top four targeted research population groups are children and youth, older adults, racial and ethnic minorities, and people with disabilities.

#### Project Themes

**Fig. 6:**
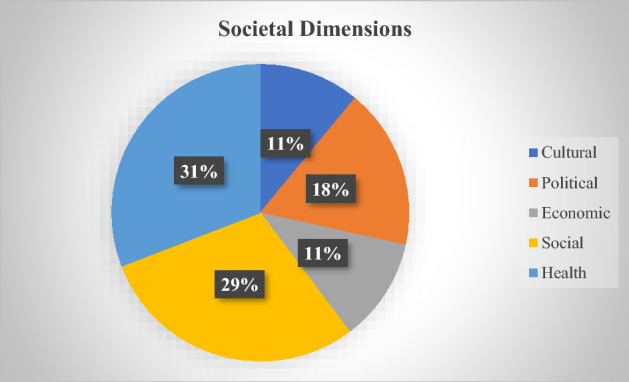
Research themes of the COVID-19-specific social scientist-led quick response disaster research projects in Canada

COVID-19 has impacted almost all societal dimensions, including cultural, political, economic, social, and health. The research project titles and related news releases generally illustrate each project’s primary societal dimension. According to Fig. 6, although most projects focused on health and related impacts, social dimensions attracted the interest of most researchers. Examples include the examination of human-animal bonds during the COVID-19 lockdown, homeschooling and family development, social media and misinformation, food security, and settlement services for immigrants and refugees. These projects specifically address the emerging and outstanding societal issues of the Canadian COVID-19 emergency response stage.

#### Collaborations

The multidisciplinary and multi-stakeholder characteristics inherent to COVID-19-driven hazards and disaster research galvanized scholars in Canada and abroad to collectively identify relevant interventions. As described above, among the 337 projects, five international projects are SSHRC funded. Among the remaining 332 domestic research projects, most (78%) focus on the researchers’ current communities. Possible contributing factors are (1) the local community is the primary field where the majority of social scientists contextualize their research ideas (Allman 2015); and (2) COVID-19’s rapid progression triggered tremendous public health restrictions during the emergency response stage (that is, social distancing, lockdown, and curfew), limiting researchers’ ability to conduct in-person field research outside their primary communities. Although interprovincial collaborative projects only represent 22% of total funded projects, these cross-provincial partnerships promote cross-jurisdiction knowledge mobilization and translation.

#### Canadian Features

The Tri-agencies accept funding applications written in both official languages of Canada (English and French). Currently, a decreasing number of French applications in each grant competition has propelled the Tri-agencies to develop related strategies to ensure equitable access to funds in both official languages (CIHR 2021b). Table 3 shows that 62 projects are written in French (61 projects funded by SSHRC and one supported by CIHR), representing 18.4% of the total awarded projects.

**Table 3:** Canadian features of the COVID-19-specific social scientist-led quick response disaster research projects in Canada

	SSHRC	CIHR	Total
Project proposals written in French	61	1	62 (18.4%)
Indigenous research project proposals	8	1	9 (2.7%)

*CIHR* Canadian Institutes of Health Research, *SSHRC* Social Sciences and Humanities Research Council

Furthermore, supporting aboriginal research and training has been prioritized in the Canadian federal research granting agencies’ 2019–2022 strategic plan (Government of Canada 2019). The Tri-agencies also established several rounds of Indigenous-specific proposal calls to encourage multidisciplinary interventions and to comprehensively address the diverse impacts of COVID-19 on aboriginal communities in order to develop responsive solutions (CIHR 2021c). This case study identifies nine funded research projects that examined aboriginal issues (eight SSHRC-funded projects and one CIHR-funded project). These projects covered a wide range of Indigenous research topics, such as Indigenous culture and resilience, Indigenous community economic recovery, Indigenous social development, and mental health. One of the eight SSHRC-funded projects was led by a French principal investigator, and the research application was written in French.

## Challenges

The findings above briefly delineate Canada’s first COVID-19-specific social scientist-led QRDR, including: (1) the demographic structure of the social science research workforce (typology, geographic locations, primary disciplines, and educational backgrounds), highlighting the diverse characteristics of social sciences researchers, and uneven research development across Canada; and (2) the characteristics of the research projects (research subjects, project themes, collaborations, and Canadian features), emphasizing the need for galvanizing cooperation and focusing on unique Canadian contexts. The data curation, analysis, and synthesis processes unveiled the following challenges, reflecting potential research outcomes and limitations. Based on these identified challenges and limitations, this section provides recommendations for improving QRDR in Canada and internationally.

First, as the official sources for this case study provided somewhat limited information, the resultant lack of complete details has prevented further analysis. For example, SSHRC-funded projects represent 87.5% of the total projects examined in this case study. The SSHRC award recipient list consists of researcher names and affiliated organizations, project titles, and total funding amount, excluding such essential information as project abstracts and keywords. Although CIHR-funded projects’ abstracts and keywords are publicly available (representing 12.5% of the total projects investigated in this research), this limited information may not be sufficient to identify additional critical project-related details, such as research methods, data collection instruments and protocols, and team structure and collaboration. Specifically, “methods matter,” particularly for social science-based QRDR (Peek, Champeau, et al. 2020). However, in the absence of this crucial information, this case study has failed to assess social science research methods, thus endangering other related analyses.

Second, COVID-19 has moved most daily routines to the virtual world; however, Internet-based searching and data collection do not always yield up-to-date information. When the authors used researcher names and affiliated organizations to search and access professional details from organizational websites, it was possible that these web pages had not been regularly updated or maintained. Therefore, some information collected may be out-of-date. Furthermore, as Canadian and international postsecondary educational organizations swiftly established their inter-/trans-disciplinary and international research collaborations, Canadian researchers have been increasingly (especially faculty members) conducting research and teaching outside of their original disciplines. With those researchers potentially working outside their original fields of training, web-based information gathering generates a risk of researcher primary discipline misidentification, in addition to other potentially faulty demographic variables. While affiliated organization research news might confirm some collected data, the research project descriptions are typically curtailed, jeopardizing further analysis.

Third, QRDR aims to convert perishable data into evidence-based interventions to inform research, practice, and policy making. Since this COVID-19-specific QRDR is considered the first official Canadian federal granting agency-supported QRDR, the research knowledge dissemination, translation, and mobilization process is still developing. Although granting agencies require a final report after project completion, this report may not be publicly available. Academic journal articles are the primary knowledge dissemination and mobilization avenue for most academic researchers. It may be difficult to track direct research outcomes for a designated QRDR grant through various publications. Consistent and effective knowledge dissemination and mobilization approaches are urgently needed to promote time-sensitive QRDR findings.

Focusing on the first federal QRDR initiatives in Canada, the data curation was based on the CIHR and SSHRC grant recipients. Although the Tri-agencies are the primary granting agencies for Canadian research communities, provincial government research and funding organizations, not-for-profit organizations, and other agencies across Canada also financially supported COVID-19-specific research activities; however, these projects were excluded from this case study. Hence, this case study might not represent the entire COVID-19-specific social science QRDR agenda and associated research workforces in Canada. A nuanced understanding of the COVID-19-specific QRDR, their research workforce, and their projects’ characteristics in Canada call for a cross-agency collaborative approach.

## Recommendations

In response to these challenges and limitations identified above, the following recommendations are provided to improve Canadian QRDR development:

*Recommendation 1*: Promoting information transparency, dissemination, and updates from the original resources could effectively address the aforementioned challenges.

Building a QRDR project tracking system not only assists research granting agencies in frequent evaluation and adjustment of their funding streams and related strategies, but more importantly, effectively promotes QRDR knowledge mobilization and strengthens their broad impacts on research, practice, and policy-making communities. For instance, NHC developed a webpage to distribute the QRDR reports (NHC n.d.b). This approach promotes knowledge mobilization among researchers, practitioners, policymakers, the general public, and other stakeholders. The ICLR-funded QRDR project reports are available with open access on the ICLR website (ICLR n.d.b). Ideally, the federal granting agencies could initiate a similar and even more advanced knowledge mobilization approach to address existing and mitigate future challenges.

*Recommendation 2*: Improving Canada’s hazards and disaster research workforce understanding.

In North America, the NHC has initiated several research projects to better understand the hazards and disaster research workforce in the United States and internationally. As demonstrated above, the total number of Canadian hazards and disaster researchers identified in this case study is significantly higher than what the reported SSEER project 2018 census data showed (Peek, Champeau, et al. 2020). This difference indicates a paucity of understanding in the Canadian hazards and disaster research workforce. In collaboration with various granting agencies across Canada, a national survey, as successfully utilized in the SSEER project, would narrow this research deficit and promote multi-stakeholder collaboration to better serve extreme event affected communities.

*Recommendation 3*: Enhancing multi-stakeholder cooperation.

Quick response disaster research is closely associated with community-based characteristics. A community-driven collaborative approach should be established among community stakeholders, local researchers, funding agencies, and beyond. This collaboration will promote community participatory research by using community-specific knowledge and skills to solve community-driven issues. This collaboration is critical to supporting Indigenous-specific research. Furthermore, as disasters do not respect geographic boundaries, this collaboration should be initiated at the local community and extend more broadly, developing mutual knowledge and promising strategies to build resilience and sustainability.

## Conclusion

The COVID-19-specific QRDR federal granting opportunities administrated through 2020 are foundational in developing Canadian national-level QRDR initiatives. The social science hazards and disaster research community has a long history of contributing to QRDR; however, the landscape of that community in a Canadian context remains unclear. Analyses of 337 SSHRC and CIHR-funded projects, with 1119 researchers, contribute to the initial understanding of the demographic structure of the Canadian social science QRDR workforce and their rapid response to COVID-19. Notably, from a researcher perspective, this case study describes researcher typology, geographic location, primary discipline, and educational background. Although these social science researchers represent the full spectrum of social science disciplines, their geographic locations are unevenly distributed across Canada. Furthermore, exploring research project-related characteristics reveals that social science scholars have examined a wide range of community-based urgent issues associated with vulnerable and marginalized populations. Project-level findings demonstrate a need to improve collaborations and focus on Canadian-specific distinctions (English/French and Indigenous research projects).

Emerging challenges in this case study are primarily associated with data curation, such as limited project information and research knowledge mobilization from both federal granting agencies and researcher-affiliated organizations. These challenges prevent this project from contributing fully to a comprehensive understanding of the complete Canadian social science QRDR workforce and their projects’ characteristics. Accordingly, three recommendations have been developed, promoting information transparency, dissemination, and updates; improving hazards and disaster research workforce understanding; and enhancing multi-stakeholder cooperation. These recommendations not only enhance QRDR development in Canada and beyond, but also indicate future QRDR-specific research topics.
